# Editorial: Insights in vascular physiology 2024

**DOI:** 10.3389/fphys.2025.1628173

**Published:** 2025-05-29

**Authors:** Luis A. Martinez-Lemus, Christopher Garland, Irena Levitan

**Affiliations:** ^1^ Department of Medical Pharmacology and Physiology, University of Missouri, Colombia, MO, United States; ^2^ The Vascular Pharmacology Group, Department of Pharmacology, University of Oxford, Oxford, United Kingdom; ^3^Department of Medicine, Pulmonary Section, University of Illinois Chicago, Chicago, IL, United States

**Keywords:** endothelial cells, ion channels, aging, kinases, hemodynamic forces

In this special issue, we present recent discoveries in vascular physiology, focusing on the regulation of blood flow and hemodynamic forces, ion channels, metabolism, LIM kinases and aging.

Blood flow is known to be regulated by the mechanical forces of shear stress via endothelial mechano-transduction. However, the role of stiffness of vascular smooth muscle cells (VSMCs) in the regulation of blood flow is virtually unknown. In this Research Topic, McCallinhart et al. presented a groundbreaking study “*Coronary Cytoskeletal Modulation of Coronary Blood Flow in the Presence and Absence of Type 2 Diabetes: The Role of Cofilin* ***”*** demonstrating that cytoskeleton remodeling of VSMCs in type 2 diabetes results in the softening of coronary resistance arteries, which augments coronary blood flow. Mechanistically, the authors showed that the softening of the VSMCs is mediated by the actin-binding protein cofilin, which promotes the disassembly of filamentous actin (F-actin), resulting in a loss of F-actin architecture. This mechanism is proposed to be compensatory to a decrease in coronary blood flow, a known complication of diabetes. Notably, this is in contrast to large arteries, which stiffen under diabetic conditions. This has a deleterious effect on cardiovascular function. This is the first indication that direct modulation of VSMCs’ cytoskeletal structure can regulate blood flow *in vivo*. The role of hemodynamic forces in vascular physiology is also addressed in this issue by Kuang et al. In their study “*Fundamental Equations and Hypotheses Governing Glomerular Hemodynamics”*. They presented a new mathematical model of the glomerular hemodynamics in the Hypothesis and Theory category, which helps to understand the physics governing glomerular filtration in a more holistic way. Finally, a review article by Chen et al. discussed recent advances in understanding the mechanisms by which low and oscillatory flow disrupts the endothelial barrier. The authors cover the complex interactions between the endothelial glycocalyx, the cytoskeleton and the junctional architecture, leading to a better understanding of how pro-inflammatory flow disrupts the barrier.

Potassium channels play a fundamental role in regulating arterial VSMCs and endothelial cell signaling via nitric oxide (NO) and EDH. Recent research has identified a key role for one form of the delayed rectifier K^+^ channel, K_V_1.3, in the development of intimal hyperplasia during type 2 diabetes, including in human arteries. Since low-grade inflammation is ubiquitous in T2D, the paper by Peraza et al. titled “*A sex-dependent role of Kv1.3 channels from macrophages in metabolic syndrome”* investigated whether other metabolic syndrome-related effects are ameliorated by inhibiting K_V_1.3 and demonstrated that blocking these channels had a primary effect against the infiltration of macrophages in female mice. Another form of K^+^-channel, the BK_Ca_ channel, which is found in VSMCs, not endothelial cells, was found to be linked to spontaneous calcium sparks, with activity suppressing vascular reactivity. These channels can also be influenced by NO. The study by Shvetsova et al. titled “*Dual Role of Calcium-Activated Potassium Channels of High Conductance: Facilitator or Limiter of NO-induced Arterial Relaxation?”* indicated that this is a dual effect in VSMCs, with BK_Ca_ limiting vasodilation to the NO-donor SNP in arteries stimulated with low concentrations of the vasoconstrictor methoxamine. In contrast, with higher concentrations of methoxamine BK_Ca_ activity was observed to enhance vasodilation to NO. The authors suggest that NO acts indirectly by inhibiting Ca^2+^ entry via VGCC, thereby limiting BK_Ca_ activity during low-level vasoconstriction. However, as vasoconstriction becomes more intense, the influence of BK_Ca_ increases, enhancing vasodilation to NO. BK_Ca_ channels are sensitive to voltage and calcium so it remains to be demonstrated whether VSM depolarization contributes to enhanced hyperpolarizing current and thus vasodilation.

A significant risk factor for the development of cardiovascular disease is hypertension. In pregnancy, it endangers the development of the embryo/fetus along with the health of the mother. Two prominent characteristics of hypertension are the presence of endothelial dysfunction and arterial stiffening. How these two characteristics of hypertension correlate in pregnant women with chronic hypertension or preeclampsia was revealed in the study by Kaihara et al. “*Differences between macrovascular and microvascular functions in pregnant women with chronic hypertension and preeclampsia: new insights into maternal vascular health*.” Their results indicate that an increased carotid-femoral pulse wave velocity is consistently present in both chronic hypertension and preeclampsia. Meanwhile, reactive hyperemia was positively correlated with blood pressure and plasma nitrite (a surrogate of nitric oxide) only in preeclampsia. Since carotid-femoral pulse wave velocity represents large artery stiffness and reactive hyperemia represents microvascular function, Kaihara et al. proposed the explanation that microvascular endothelial function is preserved in preeclampsia due to its earlier onset compared to that of chronic hypertension. An additional explanation is that endothelial dysfunction is not the initial driver of hypertension in preeclampsia. Further investigation of the potential explanations that Kaihara et al. provided for their results could influence the therapeutic approaches for the treatment of preeclampsia.

This issue also includes review articles on targeting NO production and on using the zebrafish as a model of aging. “*Promotion of Nitric Oxide Production: Mechanisms, Strategies, and Possibilities*” by Gonzalez et al. provided a brief but highly comprehensive overview of the targetable mechanisms for promoting NO production. The authors discussed the strategies currently used in clinical practice and potential approaches with specific limitations, such as specificity issues and a lack of large-scale clinical data. These limitations are appropriately described in their review. In “Cerebrovascular ageing: how zebrafish can contribute to solving the puzzle”, Malkinson and Henriques addressed the potentially impactful role of using zebrafish as a model for studying cerebrovascular aging. The researchers highlighted the advantages of assessing longitudinal cerebrovascular changes throughout the lifespan of the model and its capacity to image genetically modified and labeled targets in the whole zebrafish. Another important review article by Lateef et al. “*LIM kinases in cardiovascular health and disease*” provided a comprehensive review of the roles of LIM kinases, which regulate cytoskeleton dynamics, in cardiovascular cells. LIM kinases are known to be canonical substrates of small Rho-GTPases but despite accumulating evidence of their critical roles in the cardiovascular system, a comprehensive review has been lacking. Lateef et al. provided an in-depth analysis of the Research Topic.

